# Staging of gastric cancer with the Clinical Stage Prediction score

**DOI:** 10.1186/s12957-019-1589-5

**Published:** 2019-03-08

**Authors:** Kiyoaki Taniguchi, Masaho Ota, Takuji Yamada, Akiko Serizawa, Takeharu Noguchi, Kunihiko Amano, Sho Kotake, Shunichi Ito, Naoki Ikari, Akiko Omori, Masakazu Yamamoto

**Affiliations:** Department of Surgery, Institute of Gastroenterology, Tokyo Women’s Medical University, 8-1 Kawada-cho, Shinjuku-ku, Tokyo, 162-8666 Japan

**Keywords:** Carcinoembryonic antigen, Gastric cancer, Receiver operating characteristics analysis, Staging

## Abstract

**Background:**

Chemotherapy with or without surgery is the first-line treatment for stage III/IV gastric cancer, while surgery is the first-line treatment for stage I/II gastric cancer. Accordingly, it is important to distinguish between stage III/IV and stage I/II gastric cancer, but clinical staging is less accurate than pathological staging. This study was performed to develop a clinical score that could distinguish stage III/IV gastric cancer from stage I/II gastric cancer.

**Methods:**

We reviewed 2722 patients who underwent gastrectomy at our hospital from January 1996 to December 2015. As pretreatment factors potentially related to tumor stage, we assessed age, sex, tumor markers, tumor diameter, tumor location, tumor histology, and macroscopic type. Factors showing significance on multivariate analysis were used to develop the Clinical Stage Prediction score (CSP score), and a cutoff value for the score was determined by receiver operating characteristics analysis.

**Results:**

According to multivariate analysis, clinical factors associated with stage III/IV disease were elevation of the carcinoembryonic antigen level, tumor diameter ≥ 60 mm, circumferential gastric involvement, esophageal infiltration, mucinous adenocarcinoma, and macroscopic types 2–4.

The CSP score was obtained by weighting these factors according to the non-standardized β-coefficient. Receiver operating characteristics analysis indicated that the optimum cutoff value of the CSP score was 17 points. Among 1042 patients with a CSP score ≥ 17 points, 820 patients (78.7%) had stage III/IV gastric cancer. Conversely, among 1680 patients with a CSP score < 17 points, 1547 patients (92.1%) had stage I/II gastric cancer. When discrimination of stage III/IV gastric cancer from stage I/II gastric cancer by the CSP score was assessed, the sensitivity was 78.7%, specificity was 92.1%, positive predictive value was 86.0%, and negative predictive value was 87.5%.

**Conclusions:**

The CSP score can be helpful for differentiating stage III/IV gastric cancer from stage I/II gastric cancer based on pretreatment clinical factors.

## Background

In patients with stage I/II gastric cancer, a good outcome can be achieved by endoscopic resection or standard surgical treatment. On the other hand, although most patients with stage III/IV gastric cancer undergo resection of the primary tumor, the overall survival rate is only 14.9–67.1% [1, 2]. Gastrectomy with postoperative adjuvant chemotherapy is currently the standard treatment for stage III gastric cancer, but preoperative neoadjuvant chemotherapy using more potent anticancer agents shows promise of improving the outcome. While chemotherapy is the primary treatment for stage IV gastric cancer, it is expected that also performing surgery may prolong survival. If chemotherapy is accepted as first-line treatment for stage III/IV gastric cancer, while surgery is first-line treatment for stage I/II gastric cancer, it is important to make a differential diagnosis between stage III/IV and stage I/II disease. However, conventional clinical diagnosis is less accurate than pathological diagnosis. Therefore, we performed a retrospective analysis of factors used to make a clinical diagnosis before treatment and developed a Clinical Stage Prediction score (CSP score). Then, we investigated whether stage III/IV gastric cancer could be differentiated from stage I/II gastric cancer by using the CSP score.

## Methods

Between 1996 and 2015, a total of 2722 patients with primary gastric cancer, excluding patients with cancer of the remnant stomach, underwent surgery at the Tokyo Women’s Medical University Hospital (Tokyo, Japan). Our institutional review board waived the need for informed consent because this was a retrospective study. TNM categories were determined according to the Japanese classification of gastric carcinoma [3] (Table 1), which is widely used. In this classification, the depth of tumor invasion is recorded as the T category, lymph node metastasis is recorded as the N category, and the presence/absence and sites of distant metastasis are recoded as the M category.

**Table 1:** Patient characteristics

Age	63.7 ± 11.8
Sex	
M	1849
F	873
T	
M	647
SM	581
MP	283
SS	173
SE	858
SI	180
N	
N0	1498
N1	580
N2	417
N3	227
M	
M0	100
M0	2622
P1	233
P0	2485

TNM categories were determined according to the Japanese classification of gastric carcinoma [3]

Various pretreatment factors were investigated as potential predictors of tumor stage, including the age, sex, tumor markers (serum carcinoembryonic antigen (CEA) (≤ 5.0 ng/ml vs. ≥ 5.1 ng/ml) and serum cancer antigen 19-9 (CA19-9) (≤ 37 U/ml vs. ≥ 38 U/ml)), and tumor diameter (≤ 59 mm vs. ≥ 60 mm). To investigate the predictive value of tumor location and extent, the stomach was divided into thirds (upper third, middle third, and lower third) and the gastric circumference was divided into four equal parts for assessment of circumferential involvement (lesser curvature, greater curvature, anterior wall, and posterior wall; a circumferential category was also added). Extension of the tumor into the esophagus or duodenum was also assessed. Finally, the histological type and the macroscopic type (Types 0–4) were investigated. Macroscopic and histological types were determined according to the Japanese classification of gastric carcinoma [3].

### Statistical analysis

We initially investigated the association between pretreatment factors and tumor stage (I/II vs. III/IV) by univariate logistic regression analysis. Then, we entered the candidate factors identified by univariate analysis as explanatory variables for multivariate logistic regression analysis. Subsequently, the factors that predicted tumor stage were weighted according to the relative magnitude of the β-coefficient in logistic regression analysis to develop the CSP score.

Receiver operating characteristics (ROC) analysis was performed, and the cut-off value of the CSP score was calculated from the ROC curve by determining the Youden index. The accuracy of the CSP score for staging gastric cancer (I/II vs. III/IV) was evaluated in our patient cohort by the chi-square test. All analyses were performed with JMP software ver. 12 (SAS Institute, Cary, NC).

## Results

### Univariate analysis

Table 2 shows the associations between tumor stage (I/II vs. III/IV) and pretreatment factors according to univariate analysis.

**Table 2:** Analysis of preoperative factors and tumor stage

	Stage I/II	Percent	Stage III/IV	Percent	Total	Univariate analysis	Multivariate analysis
	*n* = 1769		*n* = 953			*P* value	*P* value
Age	62.0 ± 11.4		63.7 ± 11.8			0.0013	0.3454
Sex	F354/M704		F312/M630		F666/M1334	0.6327	
Tumor markers							
CA19-9	139	33.74	273	66.26	412	0.0002	0.3063
CEA	167	41.34	237	58.66	404	< 0.0001	0.0031
Tumor diameter							
≤ 59	1441	86.4	225	13.6	1666		
≥ 60	328	31.06	728	68.94	1056	< 0.0001	< 0.0001
Location/extent							
Anterior wall	283	77.11	84	2.89	3667	< 0.0001	0.5661
Posterior wall	427	77.5	124	2.5	551	< 0.0001	0.1243
Lesser curvature	705	66.89	349	33.11	1054	0.0987	
Greater curvature	304	68.93	137	31.07	441	0.0578	
Circumferential	50	16.18	259	83.82	309	< 0.0001	0.0205
Upper third	307	56	241	433.98	548	< 0.0001	0.7119
Middle third	855	69.85	369	30.15	1224	< 0.0001	0.3593
Lower third	607	63.89	343	36.11	950	0.3809	
Esophagus	39	23.64	126	76.36	165	< 0.0001	0.0611
duodenum	43	25	129	75	172	< 0.0001	0.2652
Histology							
TB1	449	82.39	96	17.61	545	< 0.0001	0.8469
TB2	436	63.01	256	36.99	692	0.2053	
por1	75	53.96	64	46.04	139	0.0061	0.7613
por2	471	52.63	424	47.37	895	< 0.0001	0.221
SIG	241	93.05	18	6.95	259	< 0.0001	0.133
PAP	56	64.37	31	35.63	87	0.9017	
MUC	29	34.12	56	67.88	85	< 0.0001	0.0225
ASQ	2	33.33	4	66.67	6	0.1924	
Macroscopic type							
Type 0	1278	98.38	21	1.62	1299	< 0.0001	0.0003
Type 1	53	62.35	32	37.65	85	0.6443	
Type 2	146	44.11	185	55.89	331	< 0.0001	< 0.0001
Type 3	241	32.01	512	67.99	753	< 0.0001	< 0.0001
Type 4	41	17.83	189	82.17	230	< 0.0001	0.0032

When 21 factors that were significant by univariate analysis were used as covariates for multivariate logistic regression analysis, the significant factors for discriminating tumor stage (I/II vs. III/IV) were tumor markers (CEA and CA19-9), tumor diameter ≥ 60 mm, macroscopic type (type 0, type 2, type 3, and type 4), mucinous histology, and infiltration of the esophagus. *Abbreviations*: *TB1* well-differentiated adenocarcinoma, *TB2* moderately differentiated adenocarcinoma, *por1* solid poorly differentiated adenocarcinoma, *por2* non-solid poorly differentiated adenocarcinoma, *SIG* signet ring cell carcinoma, *PAP* papillary adenocarcinoma, *MUC* mucinous adenocarcinoma, *ASQ* adenosquamous carcinoma

A tumor diameter ≥ 60 mm and higher levels of both tumor markers (CEA and CA19-9) were significantly associated with stage III/IV disease.

Regarding the influence of tumor extent and location, stage III/IV disease was significantly associated with tumors located on the anterior wall or posterior wall of the stomach, circumferential tumors, tumors in the upper third or middle third of the stomach, and tumors invading the esophagus or duodenum. With regard to histology, well-differentiated adenocarcinoma and signet ring cell carcinoma were associated with stage I/II disease, while mucinous carcinoma was related to stage III/IV disease. Finally, macroscopic tumor types 2, 3, and 4 were significantly associated with stage III/IV disease.

### Multivariate analysis

We employed 21 factors that were significant according to univariate analysis as covariates for multivariate logistic regression analysis. Factors confirmed to be significant for discriminating tumor stage (I/II vs. III/IV) by multivariate analysis were tumor markers (CEA and CA19-9), tumor diameter ≥ 60 mm, macroscopic type (type 0, type 2, type 3, and type 4), mucinous histology, and infiltration of the esophagus. However, the other 13 factors were not independent predictors (Table 2).

### Establishment of the Clinical Stage Prediction score

The CSP score was devised by assigning scores for the factors identified by multivariate analysis, with weighting according to the relative magnitude of the non-standardized β-coefficient. One point was assigned for mucinous histology, while two points each were assigned for elevation of CEA, circumferential involvement, and infiltration of the esophagus. Eight points were given for a tumor diameter ≥ 60 mm. Macroscopic types 2 and 4 received 10 points, while macroscopic type 3 was assigned 16 points because the relative β-coefficient was approximately twice that of the other types (Table 3).

**Table 3:** Scores of the factors for predicting the stage of gastric cancer

	Multivariate analysis *P* value	β-coefficient	Weighted score
Tumor marker			
CEA	0.0031	0.08	2
Tumor diameter			
≥ 60	< 0.0001	0.24	8
Location/extent			
Circumferential	0.0205	0.09	2
Esophagus	0.0441	0.07	2
Histology			
Mucinous	0.0284	0.04	1
Macroscopic type			
Type 0	0.0003	− 0.274	− 7
Type 2	< 0.0001	0.3	10
Type 3	< 0.0001	0.48	16
Type 4	0.00042	0.3	10

The CSP score was devised by assigning scores for the factors identified by multivariate analysis, with weighting according to the relative magnitude of the non-standardized β-coefficient

**Fig. 1:**
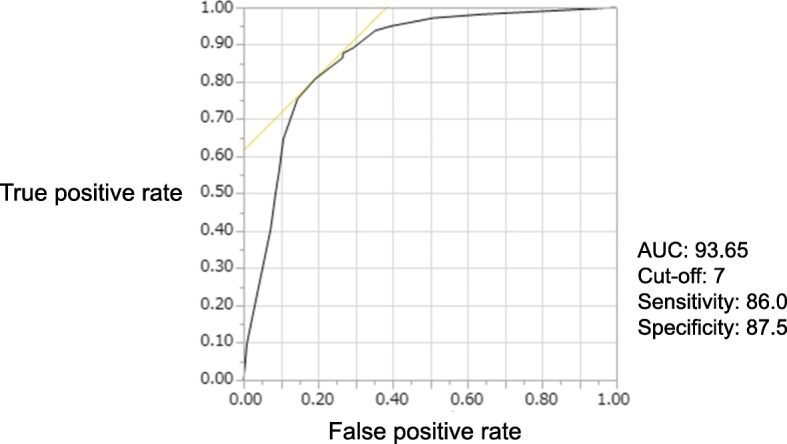
ROC curve of the CSP score. ROC analysis was performed to identify the optimum cut-off value for the CSP score, which was 17 points based on the Youden index

Then ROC analysis was performed to identify the best cut-off value for the CSP score, which was set at 17 points based on the Youden index (Fig. 1). The accuracy of a CSP score > 17 points for identifying stage III/IV disease was 78.7% (95% confidence interval [CI] 49.4–65.3%), while the accuracy of a score < 17 points for identifying stage I/II disease was 92.1% (95% CI 83.7–88.8%) When discrimination of stage III/IV gastric cancer from stage I/II gastric cancer by the CSP score was investigated, its sensitivity was 78.7%, specificity was 92.1%, positive predictive value was 86.0%, and negative predictive value was 87.5% (Table 4).

**Table 4:** Tumor stage and the CSP score

CSP score	Stage I/II	Stage III/IV	Total
> 17 points	222 pts	820 pts (78.7%)	1042 pts
≤ 17 points	1547 pts (92.1%)	133 pts	1680 pts
Total	1769 pts	953 pts	2722 pts
Sensitivity	78.7%		
Specificity	92.1%		
Positive predictive value	86%		
Negative predictive value	87.5%		

The accuracy of a CSP score > 17 points for identifying stage III/IV disease was 78.7% (95% confidence interval [CI] 49.4–65.3%), while the accuracy of a score < 17 points for identifying stage I/II disease was 92.1% (95% CI 83.7–88.8%)

For discriminating stage III/IV gastric cancer from stage I/II gastric cancer, the sensitivity was 78.7%, specificity was 92.1%, positive predictive value was 86.0%, and negative predictive value was 87.5%. *Abbreviation*: *pts* patients

## Discussion

We devised a new staging score for gastric cancer (the CSP score) by analysis of pretreatment factors in 2722 patients, and we demonstrated that this score could effectively discriminate between stage I/II disease and stage III/IV disease before initiation of treatment. It has been reported that preoperative chemotherapy may improve outcomes for stage III/IV disease, suggesting that an accurate method of predicting the stage before starting treatment could be useful. Numerous prognostic factors for gastric cancer have been reported, including the depth of tumor invasion [4], site of lymph node metastasis [4], number of metastatic lymph nodes [5–7] lymph node metastasis ratio [7–9], distant metastasis [4], results of peritoneal lavage cytodiagnosis [10, 11], tumor diameter [12], macroscopic type [13, 14], tumor location [15, 16], age [17, 18], sex [17], lymphatic invasion [19], venous invasion [20], histologic type [21], macroscopic serosal invasion [22], tumor markers (CEA and CA19-9) [23, 24], and extent of lymphadenectomy [24–26]. Most of these factors can be assessed before initiation of treatment, apart from those related to lymph nodes, lymphatic invasion, and venous invasion.

In the present study, both the tumor size and macroscopic type were confirmed to be useful for identifying advanced disease. Mucinous carcinoma was also a significant factor on multivariate analysis, which is reasonable since 90% of mucinous tumors are advanced because early cancers release most of their mucin into the gastric lumen [27].

Various modalities are employed for diagnosis and staging of gastric cancer, with each method being influenced by inherent characteristics, observation conditions, instrument performance, and differences between institutions. Therefore, clinical staging of gastric cancer has a relatively low accuracy (60–70%) compared with pathological staging [28–33]. Also, endoscopic staging is often based on clinical experience because of the lack of objective criteria for assessing the depth of invasion. While endoscopic ultrasonography (EUS) is useful, it is difficult to determine the depth of ulcerated lesions and the accuracy is no better than that of standard endoscopic diagnosis [29]. Accordingly, we investigated preoperative factors related to tumor stage and we devised the CSP score by weighting each factor to obtain a useful predictor of gastric cancer stage. The depth of invasion is assessed preoperatively by endoscopic observation, EUS, and abdominal ultrasound. It has been reported that NBI observation achieves 92% accuracy for determining the depth of invasion of early gastric cancer, but this decreases to 57–86% with white light observation and is around 74% for EUS [29].

EUS, CT, positron emission tomography, and abdominal ultrasound can all be used to assess lymph node metastasis, but the reported accuracy varies widely from 50 to 92% [30–32]. Accuracy of CT is comparatively high among these modalities, with lymph nodes > 10 mm in diameter being detectable and visible nodes likely to be metastatic [33]. However, many metastatic nodes are not enlarged, and it is impossible to predict the presence/absence of metastasis from size alone. Also, tumor progression is judged by the number of nodal metastases, but it is difficult to separate N1 (1–2 nodes involved) from N2 (3–5 nodes involved). On the other hand, imaging is useful for assessing the tumor diameter and distant metastasis, except for remote lymph node metastasis or micrometastases, while laparoscopic examination can be performed to identify peritoneal metastases [33].

Various scoring systems for gastric cancer have been reported that predict the prognosis, complications, or risks for elderly patients. A depth prediction score that separates M-SM1 disease from SM2 disease based on endoscopic findings such as tumor location, macroscopic type, and tumor size has also been reported. However, there have been few reports about diagnostic scores or methods that can judge the applicability of endoscopic treatment for early gastric cancer [34].

A risk score system has also been reported for preoperative prediction of the TNM stage of gastric cancer based on four factors: serum albumin, tumor size, and the T and N categories determined by helical computed tomography. When that score was investigated in 108 curatively resected patients, it showed poor versatility due to the large influence of the computed tomography findings [35].

While staging of gastric cancer is based on T, N, and M factors, the pretreatment factors in the CSP score are also associated with the prognosis or stage, and T, N, and M factors were specifically excluded from the CSP score to increase its objectivity and versatility.

Moreover, there have been no previous reports of a staging score that can be used to decide whether a patient should receive preoperative chemotherapy, which is the function of the CPS score developed in this study.

While we found that the CPS score was effective for discriminating between low- and high-stage disease, its sensitivity was somewhat low (78.9%). It is possible that the sensitivity of the CSP score could be improved by adding factors related to the nutritional status (albumin or prealbumin) and tumor markers (CA125 or AFP).

## Conclusion

The CSP score that we devised employs weighted pretreatment factors to differentiate stage I/II gastric cancer from stage III/IV gastric cancer. This allows discussion of all possibilities and evaluation of the optimum treatment strategy before surgery is performed.

## Abbreviations

CA19-9: Cancer antigen 19-9
CEA: Carcinoembryonic antigen
CSP: Clinical Stage Prediction
EUS: Endoscopic ultrasonography
ROC: Receiver operating characteristics
